# Supplementary material for: The species composition—ecosystem function relationship: A global meta-analysis using data from intact and recovering ecosystems
Source: PLoS One. 2020 Jul 30;15(7):e0236550. doi: 10.1371/journal.pone.0236550 (PMC7392319; doi:10.1371/journal.pone.0236550)
Supplement: Attachment — Submitted filename: renamed_a5887.docx [file pone.0236550.s005.docx]

# **PLOS ONE – Response to Reviewers**

## **General response to the Editor, and to the methodological approach suggested by Reviewer 1**

**Editor: general comments**
Review of PONE-D-19-31206: The species composition - ecosystem function relationship: a global meta-analysis using data from intact and recovering ecosystems.

This manuscript meta-analytically reviews literature on biodiversity and ecosystem function (BEF) that involved the outcomes of 25 individual restoration studies. Focusing on the methodological features of the study, the authors appropriately follow the PRISMA guidelines in reporting the findings of their meta analysis and the methods of the literature search and statistical analyses generally appear to be appropriate. There were some areas, however, where it would be beneficial to be more explicit about various methodological details of the meta-analysis. Specifically: (i) The eligibility criteria for studies being chosen for the review: the authors have considered a wide-variety of degradation types in their paper selection that is a great benefit but it is a bit questionable that 4 out of 25 studies were related to insects. Also a strong effort to find all the studies from major biomes would make the study a valuable resource for future studies in this field. One of the reviewer has pointed out that “tropical systems have severely weak represented”. (ii) The author should clarify whether all the studies included in the pooled meta-analysis have a comparable diagnostic cut-off point.

Based on the above and from the referees' comments, this paper require is a Major revisions. Please use the comments to make a thorough and careful revision.

**Introduction to rebuttal**

In this introduction we provide a detailed response to the editors points, to the reviewers comments on editors questions 1-5 and to reviewer 1’s overarching comments on the methods used in this study, as we feel reviewer 1 has not fully appreciated our objective and methodological approach to the study. Having said that, we appreciate the insights of both reviewers and the editor. We have made substantial revisions to the manuscript following these comments and believe that the paper is much improved by them. Following the introduction and overarching response in this first section, we provide specific responses to each of the reviewers comments.

On reflection, we agree that the paper is more focused and the less questionable if we focus on the species composition of plants only, and remove the four studies that report species composition of arthropods (there is a small loss of sample size, but it is compensated for by greater incisiveness in results).

If the available data are segmented by climatic biome and by continent then some regions are definitely more frequently represented than others (if the data are segmented by physiognomic ecosystem type and the ecosystem function types we used then the spread is more even). However, this reflects a bias in the literature (or in where studies are performed) rather than in our analysis. This has been observed in a number of other studies (and is now discussed, and referenced, in the revised manuscript). However we set out to find every study available objectively that might contain the requisite data for us to undertake this meta-analysis. It is not possible to search for additional papers without changing our search criteria, which have been specifically structured to be unbiased.

The insight for this paper came from the observation that ecological restoration studies provide data that allow us to test the species composition – ecosystem function relationship in natural systems, as a more meaningful alternative to the species richness – ecosystem function relationship (and we believe it has not been done in this way before). Our approach was to set clear hypotheses *a priori*, namely: Firstly, within intact natural ecosystems, what is the range of variation within species composition and within ecosystem function, and do the two differ? Secondly, among natural ecosystems that have become degraded by some means, does a general relationship exist between recovery in species composition and recovery in ecosystem function, and what is the strength and shape of this relationship?

Our approach was then to set up a very clear and objective sampling design in the form of strict search criteria for papers centred on finding papers that would have the specific data that we require to answer the hypothesis. Further, our approach was to set out our design for analysing these data *a priori*. We were interested in analysing the species composition – ecosystem function relationship in the most direct and objective way possible. We used the index that captures species composition that is most often used and is generally regarded as the least flawed (Bray-Curtis similarity index). Our approach was not to re-examine the species richness – ecosystem function relationship, which has been the subject of numerous papers (many probably more surgically designed to explore that relationship than our study). Most importantly, our approach was not to test our data against a range of indices and a range of categories and models. We view that as an exploratory approach to see what would emerge as significant (akin to a fishing expedition). Rather, we used the most parsimonious approach to answering our hypotheses, with the relevant indices, response ratios, and most major model terms decided *a priori* (although we did follow conventional meta-analysis practice in reducing a small number of potential factors in the models to the key predictor variables with the lowest AICc value).

**Search criteria for data (effort was equal for all biomes, continents, ecosystem functions etc.)**

A perennial problem with meta-analyses is that it is difficult to account for different potential sources of variation when comparing studies conducted in different parts of the globe, conducted on different species using different study methods. In this study we addressed this by first minimising any sources of variation between studies by having strict eligibility criteria for papers to be included:

- Must contain terms (RESTOR* OR REHABILIT* OR REFOREST*) AND (ECOLOG* OR ECOSYSTEM OR ENVIRON*) AND (FUNCTION* OR PROCESS* OR SERVICE*) AND (COMPOSITION OR BIODIVERSITY OR DIVERSITY).
- Only English papers and papers in ‘science technology’ research domain
- Within relevant research areas (environmental sciences ecology, biodiversity conservation, zoology, agriculture, entomology and plant science)
- All papers with relevant titles were then retained - the criteria for inclusion was broad and any paper pertaining to the restoration of ecosystems was retained.
- Abstracts of the reduced list were then read and papers retained if they met the criteria of including ecosystems under restoration and measured some aspect of species composition, together with some aspect of ecosystem function in both restored and reference sites. When it was unclear which variables had been measured, or if a reference site was used, the paper was nevertheless retained to next round.
- Papers were then read in full and all papers retained that measured both the species composition together with at least one ecosystem function in both restored and **multiple** reference sites.
- Authors of the final set of papers were contacted for raw data regarding the abundance, cover or biomass of each individual species and each measure of ecosystem function within each site reported in the paper.
- If authors were unable to provide raw data, and the data contained in the paper (together with supplementary material) were insufficient to allow analysis, the paper was excluded.

The text in the Materials and Methods has been amended, and the order of this information radically restructured, following reviewers comments. We feel the approach is now clearly set out in the manuscript.

**Accounting for variability among studies and ensuring like-for-like comparisons**

There is inherent heterogeneity in ecosystems and some may be more heterogeneous than others. Similarly restoration sites among the studies may be at different points of recovery. However, we were not comparing a species composition value to any pool or mean of ecosystem function among studies, but only to the ecosystem function values at exactly the same point of recovery in the same ecosystem in the same study. Our method therefore accounted for differential heterogeneity among ecosystems and studies:

- We took into account the variation present in each natural ecosystems (which may differ between studies) by comparing each restoration site to the range compositions found in its reference sites rather than any single value, or any universal value.
- The construction of the response ratio took into account pairwise metrics between each restoration site and each relevant reference site, then averaged this similarity to determine if the restoration site fell into the range of species compositions in reference sites, or quantify how different it was from them (see figure below).
- The modified response ratio is different from traditional response ratios, which normally include a single measure divided by the control/reference group (and sometimes weighted by SD). Our response ratio is the mean similarity between a restoration site and all relevant reference sites divided by the mean similarity within all relevant reference sites and therefore accounts for differences in natural heterogeneity among different ecosystems (i.e. more naturally heterogenous ecosystems will have greater variation among reference sites, and the denominator of the response ration will reflect this). (see figure below).

Further control of variation and of comparability was achieved by:

- Collecting raw data (rather than metrics presented in publications) to allow for meta-analysis of the data using one comparable index (Bray-Curtis similarity measure).
- At preliminary stages of data analysis and exploration, we also hypothesised time and restoration type might also play a role in accounting for variation. These factors were subsequently dropped from the analysis as no observable patterns were noted. Additionally, including these factors meant that a number of studies needed to be excluded (as these factors were not clearly defined in all studies), reducing the sample size, rendering an analysis with so many variables and their interactions reduced in power and scope.
- We hypothesised that the main sources of variation would be ecosystem type (a similar but more commonly used descriptor than biome) and ecosystem function type (and possibly their interactions) and were included as factors in the main analysis modelling in the paper.
- It is possible, however, that we were unable to account for some level of variation between studies though the above measures. For this reason ‘Paper ‘was also included as a random factor in the model to try and account for this variation separately. The presentation of both the Marginal and Conditional R^2^ value enable to reader to distinguish between the amount of variation caused by fixed alone (Marginal R^2^) as well as fixed effects combined with the random effects of paper (Conditional R^2^). The fact that both of these are low, even when ecosystem function type was taken into account (and/or ecosystems type, and/or the interaction – though including this factor did not increase R^2^), suggests that the variation is due to more than simply a variation between the studies themselves.

All data underlying the results (data points and response ratios used in the final analyses) of this study are listed without restriction in supplementary table S1.

## **Response to reviewer 1**

Reviewer #1: The authors have conducted a meta-analysis of the relationship between species diversity (via the Bray-Curtis index) and a variety of ecosystem function metrics. The topic is timely. A willingness to re-examine long-held relationships in the field is essential to continued growth, and this paper could make a valuable contribution in that sense. However, I have concerns that the analysis, as presented, lacks sufficient documentation and breadth to support the authors’ conclusions. My largest issues are:

1. Representation of study scope. The study is repeatedly presented as a wide-reaching and “global” meta-analysis, but only 25 studies were analyzed, 21 of those were plant studies (the other four were insects), and tropical systems have severely weak represented. The authors should make such limitations in taxa, region, and study size more explicit in scope of their paper and conclusions.

These are highly constructive comments and have improved the manuscript. We have removed the four studies that report on the species composition of arthropods, and the study is now a meta-analysis for data from 21 studies reporting plant species composition, which is a more focussed analysis, and the results less ‘debatable’. All models reported in the paper have therefore been re-analysed with the new (slightly smaller) data set, and all figures, table and results in the manuscript amended to reflect these results. It should be noted that the results of the main analysis was almost identical when conducted with and without arthropods. Every significant result was maintained with re-analysis and no new significance was found. The only meaningful change was the relationship in one ecosystem function type, *biotic interactions*, was noticeably stronger with the re-analysis using only plant species composition data (see results).

The manuscript contained a number of sentences which highlight that the data for these analyses are derived from a small number of studies and represent a small sample. In light of the reviewers comments this fact has been made more prominent in the revised manuscript, starting with the abstract. Sections have also been added to the Materials & Methods and Discussion in which the lack of studies in certain climatic biomes and continents are discussed. It should be noted, however, that due to the design of the literature search and the analysis (see section above and Materials & Methods), the small number of studies belie the reasonable number of samples derived for mean ecosystem values. For instance, in the main analysis 478 mean values for an ecosystem function types at restoration sites were compared with mean values for their intact ecosystem.

2. Lack of documentation on study comparability. One of the greatest challenges of a meta analysis is ensuring studies are suitably comparable in their response metric, as well as the attributes of the study systems themselves. Currently, it is difficult to evaluate how the authors addressed this challenge. Though the authors have created a standardized summary statistic, this does not address underlying qualitative differences (e.g. species, region, study methods) that might create unaccounted variation (i.e. the apples to oranges problem). This unaccounted variation could contribute to the weak correlation reported by the authors. Additionally, the authors’ summary statistic is a simple mean, and so does not account for within-study variance in the response variable. Koricheva et al.’s Handbook of meta-analysis in ecology and evolution is a useful resource for addressing these issues.

We believe the reviewer has not fully understood our method of meta-analysis. The re-structuring and some amendments to the text in the Materials and Methods, should make it clearer.

The summary statistic is not a ‘simple mean’. The analytical method is far more developed than comparing a mean values across restoration sites to a mean value across reference sites (see the section and figure above in ‘Accounting for variability…’; it is elaborated further below)

The modified response ratio is different from traditional response ratios (which would normally include a single measure divided by the control/reference group and sometimes weighted by SD). The mean similarity within a group of reference sites was calculated as the mean of all pairwise comparisons between all reference sites within that ecosystem. The mean similarity of restored sites to reference sites was calculated as the mean of all pairwise comparisons between each restored site and all of the reference sites within an ecosystem divided by the mean similarity within all the reference sites within an ecosystem, and therefore accounts for the variability between different ecosystems. In one sense, the mean similarity of restored sites to reference sites are means of means. (Using the mean similarity between restored sites and reference sites alone is problematic because it is not comparable across different studies; some ecosystems being inherently more heterogeneous than others. Failing to account for variation between reference sites, would mean that similarity in species-rich or heterogeneous ecosystems are likely to be underestimated because obtaining high values of similarity is more difficult in such systems.)

Our analysis was specifically designed to create a comparable response metric (as outlined above), and additionally to control for undue influence of studies that had many sites or many measures of ecosystem function. Studies may have had many measures of one type of ecosystem function (e.g. various soil nutrients), but after obtaining a mean for all the relevant pairwise comparisons for a measure (e.g. soil C), the means for all those soil nutrients (e.g. soil C + N + P) were again meaned to provide one value for the ecosystem function type (soil nutrient) for that ecosystem. Only this ‘mean of means’ was used in the analysis, in order to avoid the analysis being unduly weighted by numerous measures from a few studies.

The two most robust and relevant terms that may account for variance (Ecosystem function, species composition and their interactions) are explicitly included in the final models, contrary to what is alleged by the reviewer. As it is possible that we did not account for some level of variation between studies though the above measures, ‘Study’ was also included as a random factor in the model (see ‘General response…’ above for more detail).

3. Focus on a single similarity metric. A central premise of the paper is that species composition has greater impact than species richness. Why not, then, calculate both composition and richness, instead of composition alone? To me, the most powerful approach would be to compare these two measures within a common system to see if/how the results truly differ. Analyzing results from multiple metrics would make the conclusions more robust and would make better use of the power of meta analysis.

This is fair point, and doing as suggested is a completely justifiable approach. However, we did not want to present a paper that contributed yet another analysis to the species richness – ecosystem function discussion. There have been a great many papers specifically designed to explore the species richness – ecosystem function relationship. We wanted to highlight that comparisons between ecosystem function and more meaningful measures of biodiversity are possible. To inspire new directions for BEF research, and for drivers of ecosystem function in general. We certainly did not want to analyse multiple metrics, but rather test the specific hypothesis that ecosystem function may be driven by species composition, and how strongly (as it is often simply assumed to be a strong relationship). (See ‘General response…’ above for more detail).

L77-78: I would appreciate more detail on what specific components/definitions of ecosystem functioning (e.g. nutrient cycling, primary productivity, food production, etc.) the authors are referring to here and in other parts of the introduction. This would help define the scope of the paper and be more illustrative to readers not as familiar with the field.

This is a summary of relationships as appears very widely in the BEF literature. While we agree that the concept of ecosystem function is very broad, and would indeed benefit from refinement or at least categorisation, it is beyond the scope of this paper (as would be a detailed discussion of other components, e.g. species composition, used in this study). Ecosystem function is generally accepted in BEF meta-analyses without being specifically defined for each study. We followed this protocol: if a paper listed a measure as an ecosystem function we accepted it as such, but we did group them into six categories. That is set out in the paper as is the general need to refine the concept of ecosystem function.

L80-81: What breadth of regions/taxa do these studies represent?

Again, these lines are intended as a brief summary of overarching results in the literature from many studies and meta-analyses. There are many papers that have discussed summarised the contexts of these findings and they are referenced. Exploring the details of the species richness – ecosystem relationship is well covered in the literature and is not the focus of this paper. The paper now focuses on plant species composition only, and the regions in which studies are lacking is discussed in the paper.

L108: How are “intact natural ecosystems” defined? Are the systems under consideration similar in region/biome/size?

Intact natural ecosystems are explained in two sentences 17 lines further down. The revision of the Abstract provides the context for the use of ‘intact natural ecosystems’ in this paper and a fuller explanation has now also been included in the Materials & Methods. The numerous meta-analyses in the BEF literature do not attempt to source data only from similar region/biome/size. Indeed this undermines meta-analyses, and there is insufficient data in most systems globally for this. Our approach in sourcing data is clearly set out in Materials & Methods, and further discussed above.

L119-120: While I agree it is important to consider how ecosystems respond to real-world change driven by factors such as “degradation, restoration, fragmentation or climate change,” these processes likely have very different trajectories/outcomes in terms of species composition and ecosystem function. The authors might consider clarifying this, as well as what scope of altered landscapes are under consideration in this paper.

Text has been amended. The references to the “degradation, restoration, fragmentation or climate change” factors has been removed. Discussion of these factors is beyond the scope of the paper. The scope of the paper now only mentions intact and recovering ecosystems within restoration papers.

L140-143: On what evidence is this claim based? How far is “sufficiently far”?

Text has been amended, and now reads: ‘Data from restoration sites provide snapshots at arbitrary points in time, to meaningfully quantify the difference of a recovering site from their intact ecosystems in terms of species composition and ecosystem function.’ We trust this is clearer.

L171-172: Some info on studies is available in Table 2, but more summary information would be helpful. How many studies were available in each habitat category? What species/regions are represented, and what are their distributions? What types of restoration projects do the studies represent? What spatial/temporal scales do the studies cover? One of the most challenging aspects of a meta analysis is ensuring the selected studies are sufficiently comparable, so providing more summary information on the studies is essential.

All these details are provided for each data point in the supplementary table, S1. They are summarised in what is now Table 1 (previously Table 2). An additional paragraph has also been added to Materials & Methods and to the Discussion, in which this distribution and categorisation of the data is set out and discussed. The comparability of the data is explained above.

L172: What is the difference between forest and woodland?

The widely accepted definition is that in a forest tree canopies overlap often resulting in canopy layers, and in a woodland there is generally space between tree canopies usually resulting in simply a tree and ground layer. Categorisation follows the descriptions of the ecosystem type (the more commonly used descriptor than biome) given in the papers.

L196: What are some examples of an “intact ecosystem” across the different studies considered?

Text has been amended in the Abstract, Introduction and in the first paragraph of Materials & Methods to make the ‘intact ecosystem’ concept clearer.

L214: How does this equation incorporate variance in the measurements? As far as I can see, only the mean values are being used. A more informative metric might consider how much the two distributions overlap, not just how far the two means are. Otherwise, you could have two mean values that are far apart, but only because the two distributions have wide variance.

Our response ratio is the mean similarity between a restoration site and all its relevant reference sites divided by the mean similarity within all the relevant reference sites and therefore accounts for differences in natural heterogeneity among different ecosystems (i.e. more naturally heterogenous ecosystems will have greater variation among reference sites, and the denominator of the response ratio will reflect this). In doing this we allow different studies to become comparable as they are being measured against themselves, which then results in a comparable metric between studies. See above for more detail.

(Additionally, the reviewer’s premise seems incorrect: you would not have two mean values that are far apart only because the two distributions have wide variance, the variance would have to be across different ranges for the means to be far apart.)

L222-224: How similar were measures of ecosystem function across studies? (in terms of the specific metrics used, not the categories the authors assigned). This information is important to evaluate whether the measurements are truly comparable, and even when standardizing them into ratios.

The metrics and measured used in each comparison were identical. The ecosystem function of a restoration site was only compared with the same ecosystem function measure for the reference sites. The response ratio consisted of the restoration site measure over the mean of the measures for the group of reference sites. In cases where multiple measures of ecosystem function within the same ecosystem function type were provided for a restoration site, the mean response ratio across all those functions was used (rather than each measure separately) to avoid the analysis being unduly weighted by numerous measures from a few studies or from a few ecosystem function types.

All the individual ecosystem function measures are listed in S1.

L233: I am not sure that a linear model is the most appropriate way to analyze these data. My concern is that the explanatory variables, ecosystem function and species composition, are not wholly independent — both measurements are calculated for each group of reference sites. I imagine there is large variation among different groups of reference sites, and I don’t see how the model is currently accounting for this. Would it not be better to use something like a paired T-test, where the two measures are directly compared within the context of their site?

While a paired t-test would overcome this potential problem it creates more of its own. If a paired t-test was used it would either require the duplication of species composition values as multiple ecosystem function values would be paired with a single species composition value for some sites (a serious violation of the independence of samples). If only one ecosystem function measurement (either a mean, or simply one measure) were used instead, it would significantly reduce the sample size and power of the analysis. Regardless of which method is chosen, the result doesn’t change, both are still significant (with duplication t = 9.423, df = 64, p < 0.001; without duplication  t = 4.948, df = 25, p < 0.001).

L243-246: The authors should consider including additional variables in the model that might explain variation among sites, such as study, species, region, study size, etc. These factors could create variation among studies that will mask overall trends.

Study and other relevant terms (that may account for variance) are explicitly included in the model. ecosystem function, species composition and the interactions were also included in the main analysis modelling in the paper. The presentation of both the Marginal and Conditional R^2^ value enable to reader to distinguish between the amount of variation caused by fixed alone (Marginal R^2^) as well as fixed effects combined with the random effects of paper (Conditional R^2^).

At the early, exploratory stage of data analysis we also hypothesised that time and restoration type might also play a role in accounting for variation. These factors were subsequently dropped from the analysis as no observable patterns were noted.

The analytical design that ensured we were comparing only like-with-like has been explained above.

We have experienced resistance to the **findings and implications** of this study by some ecologists, which has led to undue and repetitious criticism of the **methods** of the paper, but ultimately the reviewer will simply have to accept that there is only a weak relationship between species composition and ecosystem function. The evidence is robust, even though it brings into question some foundational ecological concepts.

Table 2: Looking at the table, I am concerned about the representativeness and comparability of the data. First, the studies included in analysis are primarily plants. I believe the authors should make this clearer upfront, and structure the scope of their questions/conclusions accordingly. Second, how comparable are ants to vegetation? And what kinds of vegetation — grasses, trees? Finally, the study is described multiple places as “global,” but this is a bit of a micharacterization, as there are 10 studies from Australia, 7 from North America, 5 from Europe, 2 from Asia, 1 from South America, and 0 from Africa. This regional bias toward temperate systems should be at the very least addressed, preferably incorporated more directly in analysis, especially given the high richness of species and ecosystem services in tropical areas that is currently not being represented.

These are constructive comments that have improved the manuscript. We have removed the four studies that report on the species composition of arthropods, and the study is now a meta-analysis for data from 21 studies reporting plant species composition, which is a more focussed analysis, and the results less ‘debatable’. All models reported in the paper have therefore been re-analysed. It should be noted that the results of the main analysis was almost identical when conducted with and without arthropods.

The manuscript contains a number of sentences which highlight that the data for this analysis are derived from a small number of studies and represent a small sample. Sections have also been added to the Materials and Methods and Discussion in which the lack of studies in certain climatic biomes and continents are discussed. This a global problem in that there are fewer BEF and restoration studies from certain biomes and continents. However, in this and previous BEF studies, results have been fairly consistent among all tested ecosystem types and regions (e.g. Cardinale etal. 2006; Balvenera etal. 2006; Clarke etal 2017; Van der Plas 2019). We have also reduced the use of ‘global’ in the text.

L316: I believe the reference figure should be Fig 3, not Fig 2.

Thank you – it has been amended.

Table 3: Authors should report a full list of models run (not just the best-fitting model), as well as the AICc values. This would be fine as a supplementary table.

Tables have been added. Supplementary table S4 contains the full list of models, and S3, all AIC values.

Fig 4/5: Would it be possible to report both raw data and the modeled response in the same figure? This would make interpretation easier and take up less figure space. Similar thoughts for fig 6/7.

We found that such a figure is too confusing and is very difficult to generate sensibly, particularly as one has natural-log axes and the other linear axes.

L426-429: For a relationship like this, I would expect time to be an important factor. Among the studies included, what amount of time had the sites been degraded? How long had restoration been taking place? Can these factors be accounted for in the model?

At the early, exploratory stage of data analysis we also hypothesised that time and restoration type might also play a role in accounting for variation. These factors were subsequently dropped from the analysis as no observable patterns were noted.

We do, however, concur that time may indeed be an important factor and a useful avenue for a future study. It is therefore mentioned and discussed in the revised manuscript (Materials & Methods and Discussion). However, our study was focussed on testing whether species composition was a factor. This is a separate question, but has we believe, been robustly addressed in our paper.

L48: The authors use the Bray-Curtis index and find limited evidence for a relationship with ecosystem functioning. Would this result hold true with other composition/richness indices? How does choice of measurement influence results? Conclusions would be more robust if multiple indices were considered.

Bray-Curtis similarity indices are the most widely used in ecological studies to compare species composition as they have a number of qualities which make them numerically appropriate for comparing ecological communities (Clarke & Warwick 1994). The first quality is that if two samples have no species in common they have a similarity of 0%, and if they are identical have a similarity of 100%. The second is that it can accommodate different measures of species abundance (e.g. counts, biomass, density). Thirdly, Bray-Curtis ignores “joint absences” i.e. does not consider samples similar because they both lack a certain species.

We disagree that exploring multiple indices is a robust approach. It often leads to conducting the analysis in a trial and error manner until a pleasing result is found, and unless carefully controlled, risks finding false positives. We did not set out to write a paper that tests which indices fit with current BEF outcomes and which don’t. Rather, we are trying to direct attention to species composition as an important metric, and highlight that suitable data is available for testing such things if one looks beyond the BEF literature. We believe the more robust scientific approach is to set clear hypotheses *a priori*, and to *a priori* choose the most ecologically appropriate metric for our data,

L441: But this study did not analyze species richness, despite the seeming ease of doing so. Why not report results from both Bray-Curtis and richness, so as to compare them directly within a common analytical framework? This would make for a more robust result and take better advantage of the meta analysis capabilities.

After reflecting on this comment and that from reviewer 2, this entire paragraph has been deleted and the start of the Discussion restructured. We agree that this paragraph leads in a direction that is not the focus of the paper.

L507-509: What sizes and separation distances are represented by these sites? How many of the cited examples are plants? Are there any contrary examples that might be illustrative? I ask because (1) I would expect studies with smaller plots or greater distance between plots to have higher species turnover, and (2) I would expect plants to have higher turnover due to localized growth patterns of some species, as compared to more mobile vertebrate taxa.

This paragraph is already very detailed as relates to our study. All examples are plants. The revised text clearly refers to the vegetation type in each case. While some of the points made by the reviewer may be insightful, detailed discussion of the context and nuances of the cited studies are beyond the scope of this paper and more suited to a review paper on compositional change in intact natural communities.

Reviewer 2’s comments are the converse, viz. that this section needs shortening rather than expanding. We agree with reviewer 2 and have tightened this section substantially.

L521-525: What range of species richnesses is represented by the studies? I do not recall seeing this reported.

This text has been revised and now appears at the start of the discussion, which follows the comments of reviewer 2, and we believe improves the manuscript. These lines are a discussion of the literature, and not a comment on our results. Again we feel that climbing into many details of the cited studies are beyond the scope of this paper.

## **Response to reviewer 2**

Reviewer #2: Comments to the Corresponding author

The manuscript “The species composition - ecosystem function relationship: a global meta-analysis using data from intact and recovering ecosystems ” presents a well written study on the relationship between species composition and ecosystem function. The authors used formal meta-analysis to synthesize the outcomes of 25 individual restoration studies. At the surface, this study is about the species composition - ecosystem function relationship, but deep down, it asks a more fundamental question: Is there scientific evidence that the results from constructed BEF experiments can be transferred to real world ecosystems - as is often implied by BEF studies? Thereby, the manuscript addresses an long-standing topic and open question in ecology which makes it an immensely valuable contribution to the scientific literature around BEF studies. The manuscript underpins these questions with a sound methodological synthesis approach which is the true strength of formal meta-analyses.
The methodology is sound, the analysis is sophisticated and the conclusions drawn from the results of the study are valid. However, the results are not clearly presented and the discussion section requires much more focus. I’ll try to list some points for improvement below:

Thank you. We appreciate your understanding our aims in the paper.

The Abstract, Materials & Methods, Results and Discussion has been restructured, and the Abstract and Discussion, in particular, significantly revised.

Additionally, all models reported in the paper have been re-analysed with the new (slightly smaller, plant only) data set, and all figures, tables and results in the manuscript amended to reflect these results.

Abstract
- Overall, I find the abstract to be the weakest part of the manuscript, but it can be improved easily. For example, as someone new to the topic of restoration research I was confused how “reference sites in restoration studies” (l50) differ from “degraded but restored sites” (l55)? After going through the manuscript I think I now know what is meant here, but since PLOS One caters to a very wide and interdisciplinary audience the abstract should be more easy to access. Please rephrase accordingly.

- l56-l57 Please rephrase

- l58 The six types of ecosystem function mentioned here come somewhat out of the blue. Either mention which these six are or remove.

- l62-65 I don’t see how these conclusions can been drawn from what has been written so far in the abstract. Please change to a concluding sentence that allows the abstract to stand on its own.

- Please mention how many studies and measures were used in the meta analyses in the abstract

Unfortunately these critiques are valid. The Abstract has been comprehensively re-written. Each point mentioned by Reviewer 2 (above) is addressed, and that text amended.

Introduction
- l75ff When reflecting on the BEF field, consider swapping out the term biodiversity for species richness. Many of the the cited studies actually focus on SR.

Text amended accordingly. Terminology used is now principally ‘species richness – ecosystem function relationships’ (i.e. for all references where this is what is reported as a BEF study) except for a couple of references to the broader BEF concept.

- the literature cited in the entire paper seems a bit outdated with the most recent reference being from 2017. There have been many, also topically suitable studies been published in the last couple of years. Please bring the references up to date.

Text amended. Five references have been included for years 2017-2019, and these references have contributed to an increased focus in the paper (the result of comments from both reviewers), particularly in recent elucidation of alternative drivers of ecosystem function.

Methods:
- The first section on the “literature search” reads a bit like a textbook description of how one would conduct any meta analysis and does refer very little to the study presented here. I suggest to either move Figure 1 (PRISMA diagram) here and refer to it in the text or put some numbers in the text and move the PRISMA diagram to the appendix (I would prefer the second option). Let the reader know e.g. how many abstracts were screened, how many authors were contacted and how many studies were coded in the end, the text lacks connection to the study otherwise. I am also sure (judging from the numbers in Figure 1) you were not at able to code most studies directly from published sources – a common problem encountered when conducting meta analyses. Consider reflecting on this issue somewhere in your manuscript, either here in the methods or in the discussion section.

The Materials & Methods and Results sections have been significantly restructured following this comment. Both the method of the literature search and the results of the search are now in the Materials & Methods (and the Results are focussed only on the outcomes of the analyses). This improves the flow and sets out the entire process chronologically and in one place. Consequently the Figure 1 (PRISMA diagram) has also been move here (it cannot be placed in the supplementary section as it is required in the main text by PLOS ONE). We think the PRISMA diagram works well as part of the Materials & Methods, and that the step by step description of the search methods are important (for instance, Reviewer 1 specifically requested much detail at this level).

The relevant numbers at each stage of the search are set out in the PRISMA diagram, and more briefly in Materials & Methods. Very few studies provided sufficient data for analysis in published sources. Authors were contacted for their raw data.

- The Data Analysis section could be improved by summarizing the performed models in a table and reducing the text accordingly.

Tables have been added to the supplementary material: Table S4 contains the full list of models. We do not agree that the text should be reduced here. It is an unusual/novel analytical method and we have found that readers do not easily follow it, so the step by step layout is important ( see for instance comments of Reviewer 1).

Results:
- The Literature Search section should be merged with the Methods section, see comments above.

The Materials & Methods and Results sections have been significantly restructured following this comment. Both the method of the literature search and the results of the search are now in the Materials & Methods (and the Results are focussed only on the outcomes of the analyses).

- While informative, Table 2 is also quite wasteful regarding space and it is not very readable. Try reducing the number of rows used by introducing abbreviations and reducing repetition (e.g. only 4 out of 25 studies use something else than vegetation abundance as a species composition measure). Also, please include here the number of species composition measures and ecosystem function similarity measures per study.

Amended. This is now Table 1 in Materials & Methods. As all species composition are now measures of vegetation (permitting a column to be removed). The number of species composition and ecosystem function measures are provided in the table for each study, and in the text as totals.

- L298-306 should be integrated in the methods section as well

Text amended.

- General comment regarding all figures: they are currently of terrible quality and very difficult to read. In fact, Figs 2 and 3 are almost completely black and indecipherable. This might be caused by the submission system but should be improved for re-submission.

We believe this is caused by the submission system, which generates low resolution PDFs for circulation.

- Caption of Figure 4: include the number of studies (25) as well. This way the reader gets the full context. As the text is now it suggests a much larger scope.

Text amended (now 21 studies).

- Table 3: Please include results from all tested models not just the base and the best fitting one.

The added Table S4 contains the full list of models.

Discussion
- Overall, with 9 pages, the discussion is rather long and should be reduced by at least two and a half pages. I also suggest a restructuring of the discussion to help condensing this section: move subsection “Nature of species composition and other biodiversity relationships” to beginning. Get rid of the contrasting list and shorten the remaining subsections by half each. See below for details.

## Agreed. These comments are really constructive. The “Nature of species composition and other biodiversity relationships” section has been moved to the start of the Discussion. The ‘The species composition - ecosystem function relationship’ section reduced and the two integrated. The length of the ‘Heterogeneity within intact ecosystems’ and the ‘Differences in the relationship among ecosystem function types’ has been significantly cut. (However, some existing text has been added, primarily relating to an updated discussion of drivers of ecosystem function beyond the narrow conception in older BEF studies, and on the uneven distribution of studies and data globally).

- The contrasting list is a nice idea but does not really work. Rather write where you confirm the outcomes from expected from BEF studies and where the real world differs from BEF studies (which is actually done a bit later and would be the start of the discussion if you follow my suggestion above).

We believe this simple summary is important for readers who are not familiar with the BEF field. It sets out the major outcomes, simply, in an easily understood format, and allows readers to access the information in the manuscript at different levels of detail (not all readers will read the whole thing). We have amended the text both here and in the Introduction to make it work better, and be more pithy.

- Tighten text in l487-509.

Text amended.

- l479 - 485 I am not sure what the point is here, one could argue that the 25 studies is a low number itself. I suggest to remove this part.

Text amended. This section has be re-written to discuss the uneven distribution of studies and data around the world and trends in different ecosystem types (following comments from reviewer 1). New references have also contributed insight.

- l565-575 this largely repeats what has been already said in introduction and earlier in discussion

Agreed. Text amended - this paragraph has been removed.

- l611-613 this does not add much and could be removed

Text amended - this sentence has been removed.

- section in l592-630 can be shortend by half

Agreed. Text amended - this section has been shortened by half.
